# Identification and functional characterization of multiple haemadins and an oligomeric decorsin in the Asian land leech *Haemadipsa interrupta*

**DOI:** 10.1007/s00436-024-08404-w

**Published:** 2024-11-21

**Authors:** Christian Müller, Dana Sponholz, Céline Tolksdorf, Bernhard H. Rauch, Sebastian Kvist

**Affiliations:** 1 Animal Physiology, Zoological Institute and Museum, University of Greifswald, 17489 Greifswald, Germany; 2 Pharmacology and Toxicology, University Medicine Oldenburg, Carl Von Ossietzky University Oldenburg, 26129 Oldenburg, Germany; 3 Research Division, Swedish Museum of Natural History, 104 05 Stockholm, Sweden

**Keywords:** Haemadin, Decorsin, Blood coagulation, Platelet aggregation, Hematophagous leeches

## Abstract

Haematophagous leeches rely on a broad variety of bioactive compounds to secure a sufficient blood meal from their vertebrate prey. Both the primary (platelet aggregation) and secondary (blood coagulation) haemostasis are the main targets of action. The platelet aggregation inhibitor decorsin was first described in the North American leech, *Macrobdella decora* Say, 1824, whereas the bivalent thrombin inhibitor hirudin was originally identified in the European medicinal leech, *Hirudo medicinalis* Linnaeus, 1758. Hirudin blocks both the catalytic site and the fibrinogen-binding site (exosite I) of thrombin. Haemadin of the Indian land leech *Haemadipsa sylvestris* Blanchard, 1894, is also a highly efficient bivalent thrombin inhibitor but blocks exosite II of thrombin. So far, only the archetypal form of haemadin from *H. sylvestris* has been purified and functionally characterized, and two putative haemadins have been identified in the salivary transcriptome of *Haemadipsa interrupta* Moore, 1835, a terrestrial leech inhabiting mainly the Malayan peninsula. Here, we describe the identification of ten additional putative haemadins in the transcriptomic data set of *H. interrupta*, first generated by another study. Furthermore, we identified a putative oligomeric decorsin, which represents the first finding of this anticoagulant in a haemadipsid leech. Both the putative decorsin and a selection of haemadins were expressed, purified, and functionally characterized. The putative haemadins displayed a broad spectrum of thrombin-inhibitory potencies, whereas the putative oligomeric decorsin was indeed a weak inhibitor of platelet aggregation.

**Supplementary Information:**

The online version contains supplementary material available at 10.1007/s00436-024-08404-w.

## Introduction

Members of the leech genus *Haemadipsa* Tennent, 1859, are terrestrial leeches and inhabit tropical and sub-tropical rainforests across South and Southeast Asia (Borda and Siddall 2010). They are about 15–50 mm in length (Lai et al. 2011; Gąsiorek and Różycka 2017), possess three jaws (Sawyer 1986), are haematophagous, and mainly feed on vertebrate hosts (Fogden and Proctor 1985; Hanya et al. 2019) including humans (Stammers 1950; Wilson and Eisenberg 1982). Based on their feeding preferences, members of the genus *Haemadipsa* have been successfully applied as a source for iDNA isolation and subsequent mammalian biodiversity screening (Schnell et al. 2012; Drinkwater et al. 2021; Wilting et al. 2022; Fahmy et al. 2023). During a blood meal, they can ingest up to 13 times their initial body weight (Fogden and Proctor 1985). As described for other haematophagus leeches like *Hirudo medicinalis* Linnaeus, 1758, *Haemadipsa* leeches secrete bioactive compounds during feeding, in order to secure sufficient intake of blood. However, comparably little is known so far on the exact composition of the saliva that is secreted.

Strube et al. (1993) described haemadin from the Indian land leech *Haemadipsa sylvestris* Blanchard, 1894. Haemadin is a highly effective inhibitor of thrombin and resembles hirudin, the archetype of leech-derived thrombin inhibitors, in sequence and structure. Both factors are bivalent inhibitors of thrombin and block the catalytic site of the protease via their N-terminal segments. But whereas the N-terminal segment of hirudin comprises five amino acid residues, the respective counterpart in haemadin displays a length of nine amino acid residues. However, in both cases, an aromatic amino acid residue (tyrosine or phenylalanine) that is essential for function (Lazar et al. 1991; Strube et al. 1993; Acquasaliente et al. 2023) is located at position 3. In addition, the acidic C-terminal tail of haemadin does not interact with the exosite I (the fibrinogen-binding site) of thrombin (as is the case for hirudin) but instead blocks the exosite II (the heparin-binding site) (Richardson et al. 2000, 2002). Recent investigations revealed clear evidence that the interaction between haemadin and exosite II is only transient in nature and that the C-terminal tail prefers to bind to the exosite I of a nearby thrombin molecule instead (Acquasaliente et al. 2023).

Kvist et al. (2014) presented a salivary transcriptome of *Haemadipsa interrupta* Moore, 1935, a leech that is distributed across the Malayan peninsula. The data set was screened for the presence of sequences that may encode putative anticoagulants, and several promising candidates were identified including antistasin, guamerin, manillase, bdellin, eglin C, LDTI, and LAPP, respectively. In addition, two coding sequences of putative haemadins/hirudins were described. The first of these two sequences displayed a higher degree of sequence similarity to haemadin, whereas the second was more similar to hirudins (Kvist et al. 2014). However, none of the putative anticoagulants was functionally characterized. The first aim of the present study was hence to test whether or not the putative hirudins/haemadins of *H. interrupta* are indeed thrombin inhibitors.

The uptake and long-term storage of a blood meal by haematophagous leeches critically rely on the presence of different anticoagulants in their saliva, and inactivation or loss of the respective coding genes may have detrimental effects on survival. Consequently, the genomes of *H. medicinalis* and *Hirudinaria manillensis* Lesson, 1842, contain several copies of hirudin and hirudin-like factor (HLF) genes (Müller et al. 2016; Lukas et al. 2019). The second aim of the present study was hence to analyze whether or not the transcriptome of *H. interrupta* may contain additional sequences of putative hirudins/haemadins.

Interestingly, one of the putative haemadins of *H. interrupta* contains an RGD motif that is located within the acidic C-terminal tail of the molecule. The RGD motif is known to mediate the binding of the respective protein to cellular receptors of the integrin family (D’Souza et al. 1991), including to the glycoprotein IIb/IIIa complex on platelets (Calvete 1995). The presence of a RGD motif is a typical feature of decorsins, a type of leech-derived factor that was first described in the North American leech *Macrobdella decora* Say, 1824 (Seymour et al. 1990) and subsequently also in *Limnobdella mexicana* Blanchard, 1893 (Pfordt et al. 2022). Hirudins and decorsins share conserved structural and genomic features (Min et al. 2010; Müller et al. 2019). In contrast to hirudins, decorsins do not inhibit thrombin but are inhibitors of platelet aggregation. The RGD motif mentioned above is located between the 5th and the 6th cysteine residue within the central globular domain of decorsins, but not within the C-terminal tail as observed for the first putative haemadin of *H. interrupta*. Nevertheless, it might be possible that the factor is indeed an inhibitor of platelet aggregation. The third aim of the present study was hence to evaluate whether or not the presence of an RGD motif within the C-terminal tail of a putative haemadin facilitates the inhibition of platelet aggregation.

All hirudins, haemadins, and decorsins described so far contain only one central globular domain and are hence “monomeric” molecules. The existence of a dimeric hirudin-like molecule (named tandem-hirudin, TH) was confirmed only recently (Lukas et al. 2022), and the authors developed a model for the generation of putative oligomeric hirudin-like molecules (multiplication hypothesis). However, so far, no representative of hirudin-like molecules with more than two repeats of the central globular domain could be identified. The fourth aim of the present study was hence to evaluate whether or not *H. interrupta* may encode dimeric or even oligomeric hirudin-like molecules.

## Materials and methods

### Transcriptomic data

Transcriptomic data of *Haemadipsa interrupta* were obtained from GenBank sequence read archive number SRX246952 (Kvist et al. 2014).

### Bioinformatics tools

Basic Local Alignment Search Tool (BLAST) searches were performed using either the respective NCBI web portal (https://blast.ncbi.nlm.nih.gov/Blast.cgi) or BioEdit v7.2.5 (Hall 1999) and customized settings (word size and expected threshold values) for search algorithm parameters.

Multiple sequence alignments were generated using either ClustalX 2.1 (Larkin et al. 2007) or the CLC Sequence Viewer software package v8.0 (QIAGEN, Aarhus, Denmark) at default settings. Alignments were exported as msf-files and further processed using GeneDoc v2.7 (Nicholas and Nicholas 1997). Signal peptide sequences were predicted using the Phobius web portal (Käll et al. 2007) and SignalP6.0 (Teufel et al. 2022).

### Gene synthesis

cDNA fragments of putative haemadins/hirudins and the oligomeric decorsin were generated using the gene synthesis service of Synbio Technologies (Monmouth Junction, NJ, USA).

### Amplification and cloning of haemadin/hirudin and decorsin cDNAs

For the amplification of putative haemadins/hirudins and the oligomeric decorsin, primers were derived from the respective transcriptome database sequences. A list of all primers that were used in the study is provided in Supplementary Information Table S1. PCR reactions were performed using Q5 high-fidelity DNA polymerase (New England Biolabs, Frankfurt a. M., Germany), fragments of relevant sizes were purified and cloned, and their sequences were determined.

### Expression, purification, processing, and quantification of putative haemadins/hirudins and decorsins

The detailed protocol to clone cDNAs as well as to express, purify, and quantify the respective putative haemadins/hirudins and decorsin was described in a couple of recent publications (Müller et al. 2016, 2020a, b; Pfordt et al. 2022). Briefly, we applied an expression and purification system developed by QIAGEN (Hilden, Germany). The pQE30Xa vector encodes a factor Xa protease recognition site located between the His-tag coding region at the 5′ side and the multiple cloning site at the 3′ side. A subsequent factor Xa protease treatment cleaves off the His-tag and results in a recombinant protein that is devoid of any vector-derived amino acid residues at the N-terminus. Molar concentrations of final protein solutions were calculated by dividing the absorbance at 280 nm by the molar absorption coefficient according to the equation *ε* = (nW × 5500) + (nY × 1490) + (nC × 125) (Gill and von Hippel 1989; Pace et al. 1995). Representative gel images that illustrate the entire expression and purification process for three recombinant factors are provided in Supplementary Information Fig. S1.

### Blood coagulation assays

To verify the thrombin-inhibitory potency of putative haemadins/hirudins, we performed the thrombin time test (TT; reference range 16.8–21.4 s) using a BFT II analyzer (Siemens Healthcare, Erlangen, Germany). All steps were carried out according to the manufacturer`s instructions. Protein samples were diluted with buffer to reach final concentrations in the reaction assays of 3.2 µmol/l or 0.32 µmol/l, respectively. The desired amount of substrate was directly transferred into the cuvette immediately before the plasma was added. Dade® Ci-Trol® 1 (Siemens Healthcare, Erlangen, Germany) was used as standardised human plasma. The incubation of reaction mixtures was carried out at 37.4 °C. Measurements that exceeded 300 s were stopped and considered as complete inhibition of clot formation. Coagulation tests were performed in three technical replicates.

### Platelet aggregation assays

All assays were performed with human blood samples that were obtained from a healthy volunteer (CM) after written informed consent and approval from the institutional ethics committee. The blood collection, blood preparation, and subsequent experiment followed the procedure as already described in Lukas et al. 2019 and Pfordt et al. (2022) with few modifications. Briefly, 10 ml of venous blood was taken from the antecubital vein using an S-Monovette® (Sarstedt, Nürnbrecht, Germany) prefilled with citrate buffer. The first centrifugation step of the blood collection tube was performed at 200 g for 20 min. After centrifugation, the supernatant (platelet-rich plasma, PRP) was transferred, and the remaining blood was centrifuged again for 10 min at 2000 g. The supernatant was dedicated as platelet-poor plasma (PPP), transferred, and used as a reference value for maximal platelet aggregation. Measurements were performed using a TA-8 V aggregometer (Diagnostica Stago S.A.S., Asnières-sur-Seine, France). The snake venom-derived platelet aggregation inhibitors tirofiban and eptifibatide (Sigma-Aldrich, Taufkirchen, Germany) were used as positive controls. PRP was pre-incubated with the respective test and control compounds (final concentration 3.2 µmol/l) or buffer for 1 min at 37 °C. For the measurement, PRP was then transferred into cuvettes and stimulated with ADP (200 µmol/l; Hart Biologicals, Hartlepool, UK; final concentration 5 µmol/l) after 1 min of runtime. The final volume of each cuvette was 250 µL of diluted PRP. All experiments were performed at 37 °C over a time period of 10 min. Maximal aggregation in percentage and maximum slope of the curve in percentage per minute were calculated as quantitative output parameters (Zhou and Schmaier 2005). Platelet aggregation tests were performed in two to four technical replicates.

## Results

### Identification of putative haemadins/hirudins in *H. interrupta*

We thoroughly re-investigated the raw reads of *H. interrupta* (SRX246952) and confirmed the observations of Kvist et al. (2014). Furthermore, we were able to identify the coding sequences of ten additional putative haemadin/hirudin sequences. The lengths of all 12 putative haemadins/hirudins (named Hint_V1–V12) varied considerably between 39 and 66 amino acid residues (without signal peptides), and the isoelectric points (pI values) covered a range between 3.97 and 8.60. A summary of biochemical properties and the N-terminal sequences of all factors are provided in Table 1.

**Table 1:**
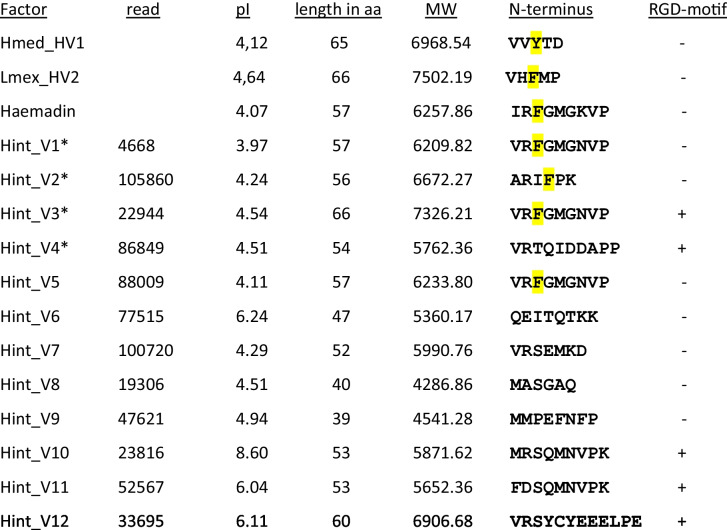
Molecular and structural properties of hirudins of hirudins of *H. medicinalis* (Hmed_HV1) and *L. mexicana* (Lmex_HV2), haemadin of *H. sylvestris*, and of putative haemadins of *H. interrupta* (Hint_V1–12)

The read numbers refer to the respective reads in SRX246952. The sequences of the N-termini are provided, and the presence ( +) or absence ( −) of the RGD motif within the C-terminal tail is indicated. The aromatic amino acid residues tyrosine (Y) or phenylalanine (F) within the N-termini are highlighted in yellow. Asterisks indicate the factors that were selected for further functional analyses

*pI* isoelectric point, *MW* molecular mass

**Fig. 1:**
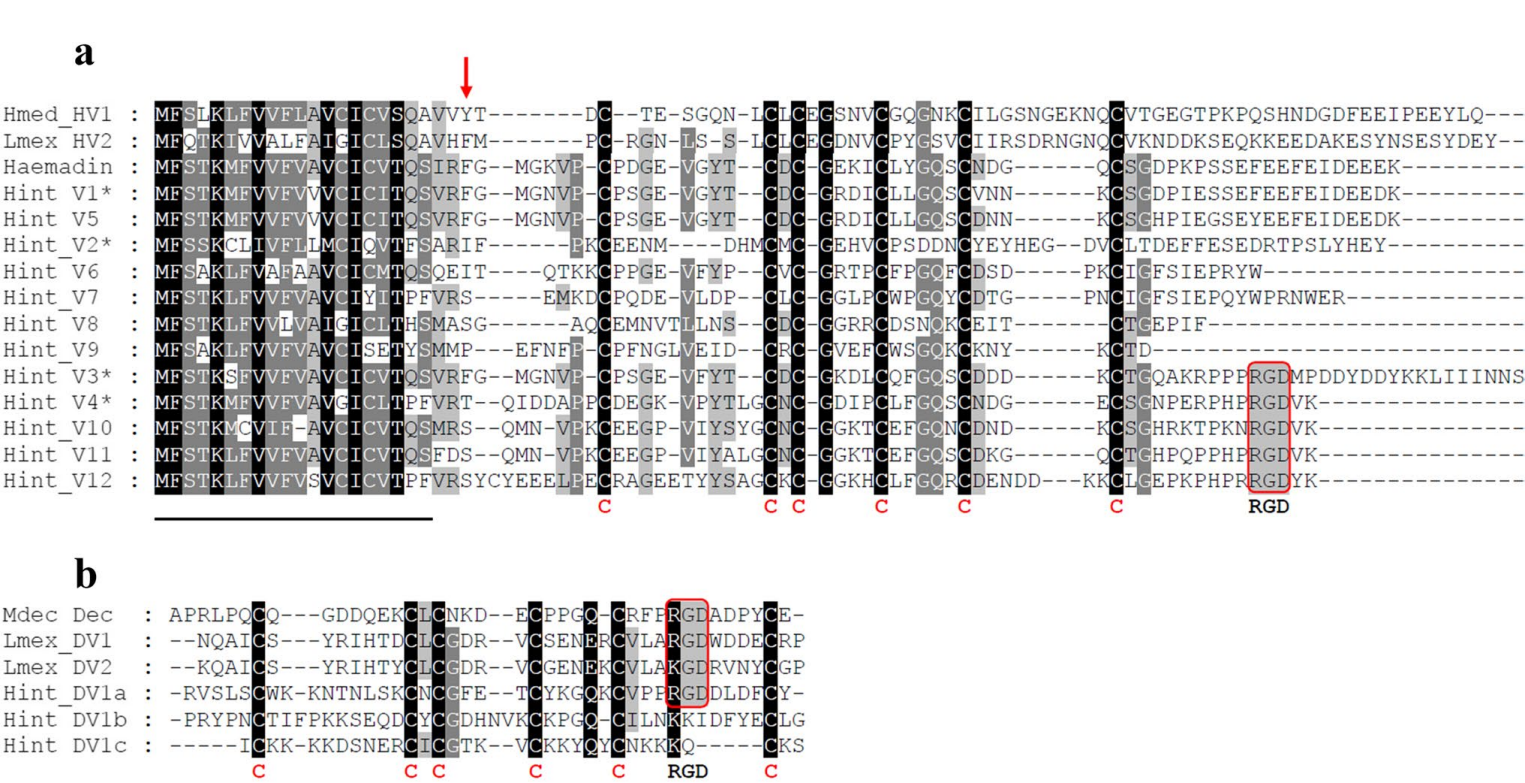
Multiple amino acid sequence alignments. **a** Hirudin variants HV1 of *H. medicinalis* (Hmed_HV1) and HV2 of *L. mexicana* (Lmex_HV2), haemadin of *H. sylvestris* and putative haemadin variants Hint_V1–12 of *H. interrupta*. **b** Decorsin of *M. decora* (Mdec_Dec), decorsin variants 1 and 2 of *L. mexicana* (Lmex_DV1 + 2), and the three globular domains of the putative oligomeric ornatin of *H. interrupta* (Hint_DV1a-c). A black background indicates fully conserved residues; a gray background indicates partially conserved residues. The six conserved cysteine residues giving rise to the three-dimensional structure are marked in bold and red. The canonical RGD/KGD motif in decorsins is marked in bold and boxed. The signal peptide sequences are underlined. Asterisks indicate the factors that were selected for further functional analyses. Abbreviations are used according to the IUPAC code

The degrees of amino acid sequence identity/ similarity/gaps between haemadin of *H. sylvestris* and the putative haemadins/hirudins of *H. interrupta* varied between 77/84/0% (Hint_V1) and 18/30/28% (Hint_V2 = contig_c1987_1). Within the putative haemadins/hirudins of *H. interrupta*, Hint_V1 and Hint_V5 were almost identical (94/96/0% sequence identity/similarity/gaps), whereas Hint_V2 was distantly related to all the other factors (12–20/20–28/13–44% sequence identity/similarity/gaps). A multiple sequence alignment of all putative haemadin/hirudin variants of *H. interrupta* is shown in Fig. 1a; a compilation of all pairwise sequence comparisons can be found in Supplementary Information Table S2. Five factors (Hint_V3 = contig c7044_1, _V4, _V10, _V11, and _V12) contained the RGD motif within the C-terminal tail (see Fig. 1a). Notably, three factors (Hint_V1, _V3, and _V5) contained a phenylalanine (F) residue at position 3 of the mature protein (marked with an arrow in Fig. 1a). An aromatic amino acid residue (tyrosine, phenylalanine or tryptophan) at position 3 is essential for binding of haemadin/hirudins to the active center of thrombin (Lazar et al. 1991; Strube et al. 1993; Acquasaliente et al. 2023).

**Fig. 2:**
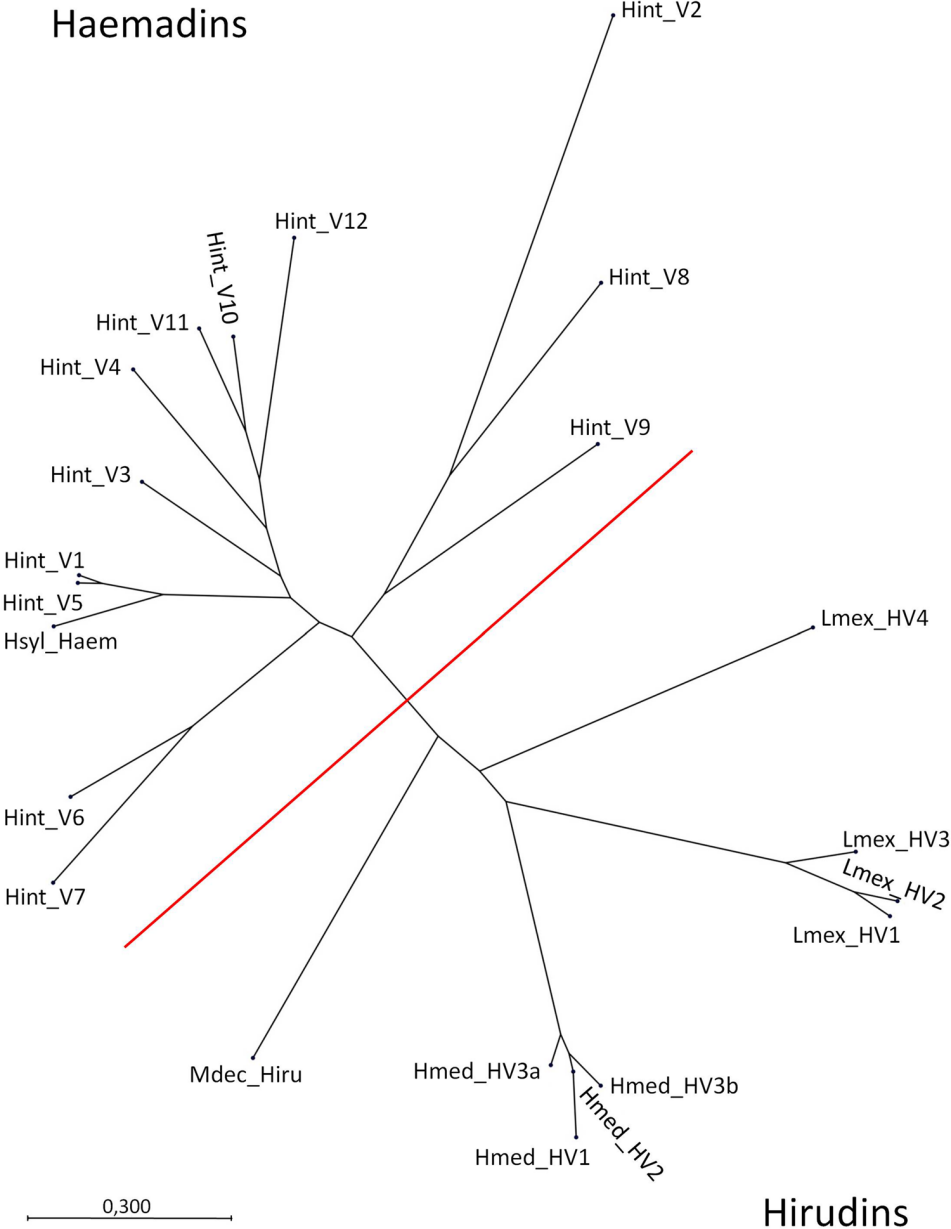
Unrooted phylogenetic tree based on amino acid sequences including the signal peptides of haemadins and hirudins generated using the neighbor-joining algorithm. Hsyl_Haem = haemadin of *H. sylvestris*; Hint_V1–12 = putative haemadins of *H. interrupta*; Mdec_Hiru = hirudin of *M. decora*; Lmex_V1–4 = hirudin variants 1–4 of *L. mexicana*; Hmed_HV1-3 = hirudin variants 1–3 of *H. medicinalis*. The scale bar indicates substitutions per site. The red bar illustrates the separation of the haemadin and the hirudin clades

To further evaluate the relationships between the putative haemadins/hirudins of *H. interrupta* we performed phylogenetic investigations and included haemadin of *H. sylvestris* and hirudins of *H. medicinalis*, *M. decora* and *L. mexicana* into our analysis. The resulting unrooted phylogenetic tree displays a clear separation between hirudins and (putative) haemadins of *H. interrupta* including Hint_V2 (= contig_c1987_1) (see Fig. 2). Interestingly, the RGD motif containing haemadin sequences (Hint_V3, _V4, and _V10-12) nicely group together.

### Identification of a putative oligomeric decorsin in *H. interrupta*

To our surprise, the re-analysis of the data revealed not only the presence of a great diversity of putative haemadin sequences in the salivary transcriptome of *H. interrupta*, but also a sequence of a putative oligomeric decorsin. As already stated in the Introduction section, decorsins are inhibitors of platelet aggregation and contain an RGD motif that is located between the 5th and 6th cysteine residues within the central globular domain. *H. interrupta* expresses a putative decorsin (named Hint_DV1) that is composed of a short N-terminus of five amino acid residues in length followed by three repeats of the (central) globular domain (named Hint_DV1a-c, respectively) and an elongated C-terminal tail. The complete amino acid sequence of Hint_DV1 and a schematic drawing that illustrates its putative secondary structure are provided in Supplementary Information Fig. S2. The composition of Hint_DV1 with three repeats of the (central) globular domain leads to the presence of 18 cysteine residues within the molecule. Hint_DV1 can be designated as an oligomeric decorsin, and this is to our knowledge the first description of a member of the hirudin superfamily that is composed of more than two repeats of the central globular domain. Interestingly, only the first globular domain repeat (Hint_DV1a) contains an RGD motif between the 5th and the 6th cysteine residue (see Fig. 1b and Supplementary Information Fig. S2). The degrees of sequence identity/similarity/ gaps between the decorsins of *M. decora*, *L. mexicana*, and Hint_DV1a of *H. interrupta* are rather low and within a range of 24–30/34–38/9%, respectively. A multiple amino acid sequence alignment of decorsins and the three repeats of the (central) globular domain of Hint_DV1 (Hint_DV1a-c) is shown in Fig. 1b, a compilation of all pairwise sequence comparisons can be found in Supplementary Information Table S3.

### Functional characterization I: thrombin inhibition

**Fig. 3:**
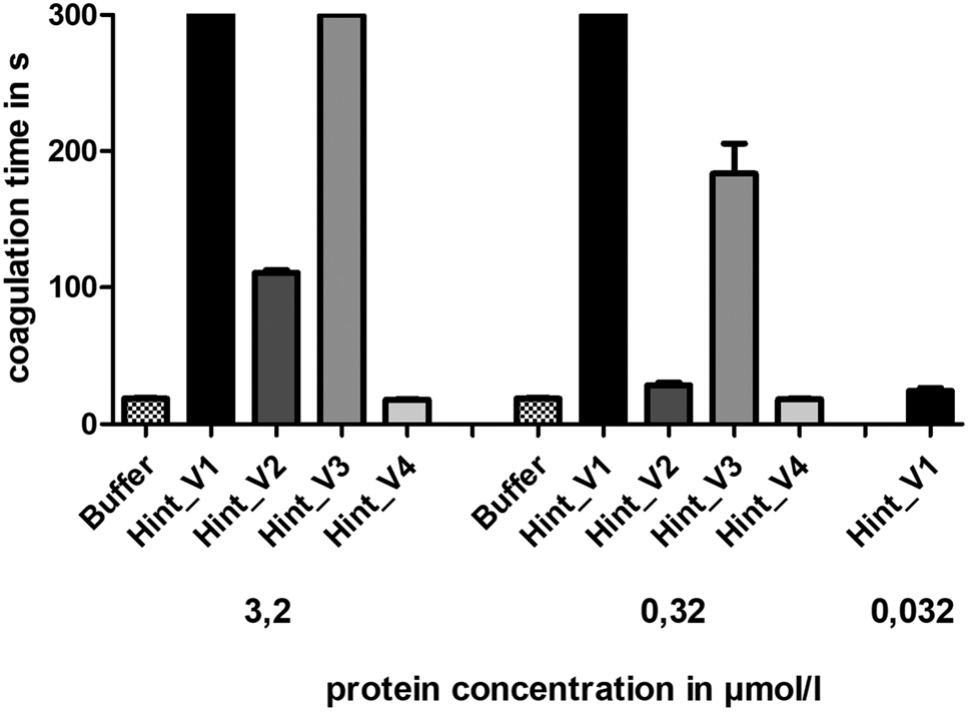
Standard blood coagulation assays of putative haemadin variants of *H. interrupta* (Hint_V1–4) using the thrombin time test (TT). Results are the mean of three independent measurements

Some, but not all putative haemadins of *H. interrupta* displayed structural and biochemical features that are mandatory for thrombin inhibition. Of special importance in that context is the composition of the N-terminus with a special emphasis on the first three amino acid residues, the acidic composition of the C-terminal tail, and an overall isoelectric point of about 4. Based on these criteria (see Table 1 for details), we decided to functionally characterize the following selection of putative haemadins of *H. interrupta*: Hint_V1 (most similar to haemadin), Hint_V2 (= contig c1987_1), Hint_V3 (= contig c7044_1, RGD motif)), and Hint_V4 (RGD motif). All factors were successfully expressed, purified, processed, and tested in the thrombin time test. Both Hint_V1 and Hint_V3 were strong inhibitors of thrombin, whereas Hint_V2 displayed a weak, but measurably inhibitory potency. Hint_V4, however, did not inhibit the activity of thrombin (see Fig. 3).

### Functional characterization II: platelet aggregation

**Fig. 4:**
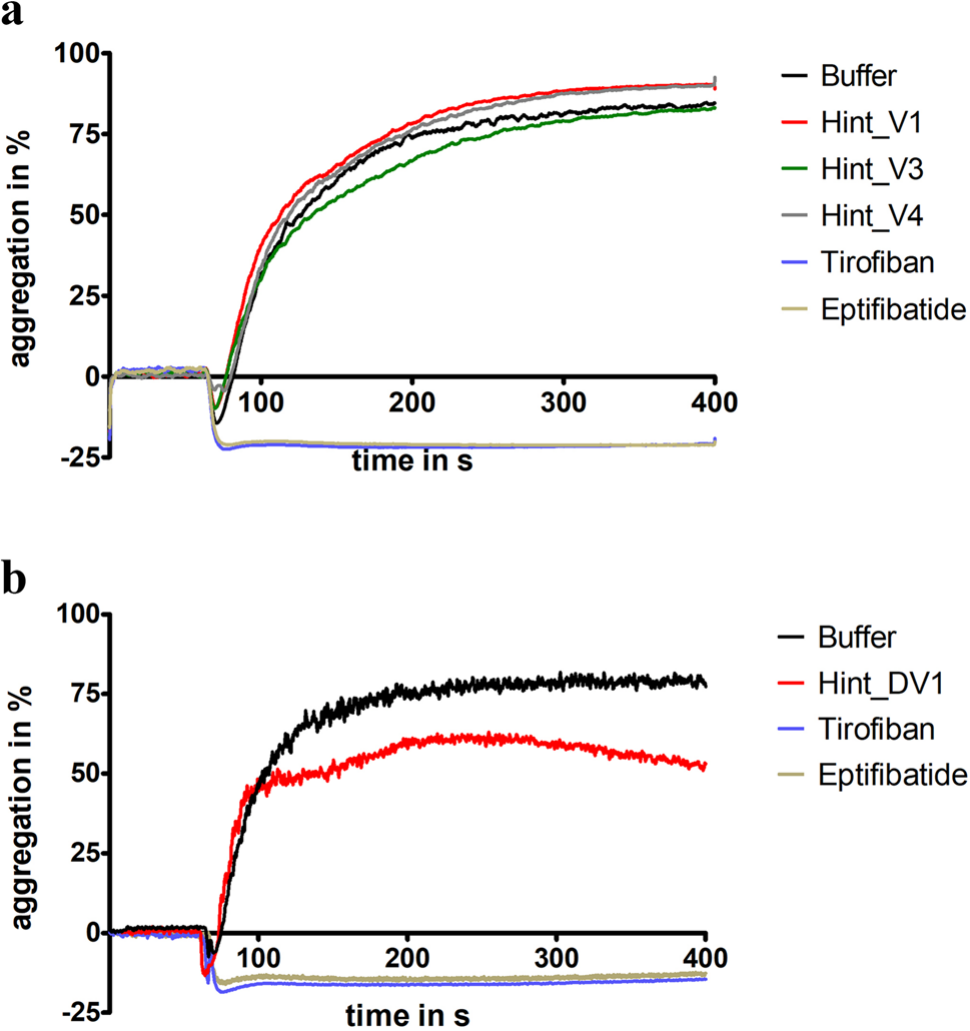
Standard platelet aggregation assays of putative haemadin variants and the putative oligomeric decorsin of *H. interrupta*. **a** Platelet aggregation curves of putative haemadins Hint_HV1, _V3, and _V4. **b** Platelet aggregation curves of the putative oligomeric decorsin Hint_DV1. Eptifibatide and tirofiban were used as positive control substances for the complete inhibition of aggregation; buffer was used as a negative control. Platelet aggregation was induced by the addition of ADP to a final concentration of 5 µmol/l. Results are the mean of two independent measurements

To measure the inhibition of platelet aggregation we selected the haemadin variants Hint_V1 (no RGD motif), Hint_V3, and Hint_V4 (both with RGD motifs) of *H. interrupta*. The snake venom-derived platelet aggregation inhibitors tirofiban and eptifibatide were used as positive controls. As can be seen in Fig. 4a, none of the haemadins of *H. interrupta* displayed an inhibitory effect on platelet aggregation.

In addition, we also expressed, purified, and tested the putative oligimeric decorsin of *H. interrupta*. The inhibitory effect of Hint_DV1 on platelet aggregation was less pronounced compared to tirofiban and eptifibatide, but clearly detectable (see Fig. 4b).

## Discussion

*Haemadipsa interrupta* is a terrestrial leech that feeds on the blood of vertebrate hosts. Unsurprising, the analysis of a salivary gland transcriptome of the leech revealed the expression of a broad variety of putative anticoagulants, among them two representatives of the haemadin/hirudin-type of thrombin inhibitors (Kvist et al. 2014). However, a thorough re-investigation of the data set uncovered the presence of several additional putative haemadins/hirudins in addition to a putative oligomeric decorsin. The variety of putative anticoagulants in *H. interrupta* is obviously much higher than originally assumed. In the present study, we focused on haemadins/hirudins and did not search for other types of anticoagulants (e.g. antistasins), but it might well be that our observations represent only the tip of the iceberg. Our results on the great diversity of putative haemadins/hirudins in *H. interrupta* are in line with similar observations in other haematophagous leeches including *H. medicinalis* (Müller et al. 2016), *Poecilobdella* (*Hirudinaria*) *manillensis* Lesson, 1842 (Müller et al. 2017), *Whitmania pigra* Blanchard, 1887 (Müller et al. 2022), and *Limnobdella mexicana* (Pfordt et al. 2022) and hold true also for other classes of major anticoagulants in leech saliva like the hirustasin/antistasin family (Iwama et al. 2021; Liu et al. 2023). However, a solely sequence-based prediction of a function is highly problematic and may be misleading. For that reason, we performed both coagulation and platelet aggregation assays on a selection of the putative haermadins/hirudins in *H. interrupta* to verify their biological function.

The archetypes of haemadin and hirudin are both highly effective bivalent inhibitors of thrombin (Strube et al. 1993; Bichler and Fritz 1991), yet their modes of action are slightly different. Whereas the acidic C-terminal tail of hirudin blocks exosite I of thrombin (Grütter et al. 1990), the tail of haemadin interacts with exosite II (Richardson et al. 2000). Both factors block the catalytic centre of thrombin by inserting their N-termini into the active site cleft. Of special importance in that context are the first three amino acid residues, especially the third residue has to be an aromatic amino acid residue (Lazar et al. 1991; Strube et al. 1993; Acquasaliente et al. 2023). Notably, only three out of the 12 putative haemadins/hirudins of *H. interrupta* (namely Hint_V1, _V3, and _V5, see Table 1) contain a phenylalanine residue at position 3, Hint_V2 at position 4. We also found that Hint_V1 and Hint_V3 were highly potent thrombin inhibitors, whereas the inhibitory effect of Hint_V2 was much reduced, but still observable. Hint_V4 contains a threonine residue at position 3 and displayed no inhibitory potency at all. A comparable substitution of phenylalanine (or tyrosine) with threonine at position 3 in hirudin variant HV1 of *H. medicinalis* increases the Ki value of the respective recombinant factor by more than three orders of magnitude (Lazar et al. 1991). We have not yet functionally tested the remaining putative haemadins/hirudins of *H. interrupta*, but based on the structural and biochemical features of the respective factors (see Table 1) and the degrees of sequence identities/similarities (see Table S1), it seems reasonable that only Hint_V5 is a thrombin inhibitor. The biological functions of Hint_V6-V12 remain obscure; they may not be haemadins (in a narrow sense) but rather haemadin-like factors (a comparable term was recently introduced for the hirudin-like factors, HLFs; Müller et al. 2019).

Haemadin and hirudin do not only differ in their binding modes to thrombin (see above), but also in the length of their N-termini: haemadin comprises nine amino acid residues in front of the first cysteine residue, hirudin only five. The lengths of the respective N-termini of Hint_V1-V12 differ between six and 12 amino acid residues (see Table 1 for details). The highly potent thrombin-inhibitory variants Hint_V1 and Hint_V3 comprise nine residues (identical to haemadin), the less effective variant Hint_V2 has six residues, and the inactive variant Hint_V4 has 10 residues. It would be interesting to see whether or not a modification of the N-terminus (e.g., removal of the residues 6–9 in Hint_V1) would alter the thrombin-inhibitory potency. Acquasaliente et al. (2023) constructed a deletion mutant of haemadin, named haemanorm, a 29-amino acid peptide containing the amino acid residues 1–9 and 41–57 of haemadin. Haemanorm is a highly effective bivalent thrombin inhibitor that blocks both the active site and, surprisingly, the exosite I of thrombin (Acquasaliente et al. 2023).

Interestingly, neither the variable lengths of the N-termini in Hint_V1-V12 nor the presence/absence of the RGD motif within the C-terminal tail altered the allocation of the factors in phylogenetic analysis: all haemadins/haemadin-like factors formed a clade that was clearly separated from the hirudin clade (see Fig. 2). The question whether or not the haemadins/haemadin-like factors nonetheless belong to the hirudin superfamily crucially depends on their gene structures. All hirudins and hirudin-like factors analyzed so far share a common gene structure that is composed of four exons and three introns (Scacheri et al. 1993; Müller et al. 2017, 2022). The gene structures of haemadins and haemadin-like factors remain to be determined. It is, however, reasonable to assume that their genes share the same basic structure.

Decorsins (and the structurally and functionally equivalent ornatins; Mazur et al. 1991) are inhibitors of platelet aggregation and belong to the hirudin superfamily as well (Müller et al. 2019). They are characterized by the presence of RGD (or in some cases KGD; see Fig. 1b) motifs that are located within the central globular domains between the 5th and the 6th cysteine residue at the apex of an exposed hairpin loop and facilitate the binding to integrin receptors (Krezel et al. 2000). Five out of the 12 haemadins/haemadin-like factors of *H. interrupta* (Hint_V3, Hint_V4, and Hint_V10-12) contain RGD motifs that are located within the C-terminal tail (see Fig. 1a and Table 1). The tail is highly flexible in solution (Folkers et al. 1989), and the RGD motif within the tail may hence come in contact and bind to integrin receptors as well. However, none of the RGD motif-containing factors displayed any evidence of an inhibitory effect on platelet aggregation (see Fig. 4a). It might be that the binding of an RGD motif located in the tail region of the protein is of low affinity and thus unstable, and/or those additional structural components within the central globular domain are necessary for binding.

In contrast, the oligomeric decorsin Hint_DV1 displayed a weak but detectable inhibitory effect on platelet aggregation. The inhibitory effect was much less pronounced compared to both the positive controls tirofiban and eptifibatide and the decorsins of *L. mexicana* and *Haementeria vizottoi* Castro, 1971 (Pfordt et al. 2022). However, our results represent the very first evidence at all for the biological activity of a hirudin-like factor that is composed of more than one central globular domain. Tandem hirudin (TH) of *P. manillensis* was described just recently (Lukas et al. 2022), but no biological function could be assigned to the factor. Furthermore, the identification of Hint_DV1 supports the hypothesis of hirudin multiplication (Lukas et al. 2022). Again, it would be highly interesting to determine and analyze the gene structure of Hint_DV1. The comparably low inhibitory potency of our preparation of Hint_DV1 could well be due to the imperfect formation of the secondary structure of the protein during heterologous expression in a bacterial host. The structures of the central globular domains of all hirudins, HLFs, haemadins, and decorsins entirely depend on the correct formation of the three disulfide bonds. The folding of hirudin is a complex process (Chatrenet and Chang 1993) and perturbations may result in the formation of scrambled and hence inactive isoforms (Chatrenet and Chang 1992; Chang et al. 1995). The situation is even more complicated in the case of Hint_DV1 which contains 18 cysteine residues instead of six and requires the correct formation of nine disulfide bonds.

The identification of such a broad variety of haemadins and haemadin-like factors in addition to an oligomeric decorsin in the land leech *H. interrupta* certainly expands our knowledge of that particular class of leeches and may also help to better understand the complex biology of haematophagous leeches in general.

## Supplementary Information

Below is the link to the electronic supplementary material.

Supplementary file1 (PDF 2904 KB)

## Data Availability

Original sequence data are deposited in GenBank under sequence read archive number SRX246952. The respective read numbers are provided within the manuscript.
