# Supplementary material for: Identification and functional characterization of multiple haemadins and an oligomeric decorsin in the Asian land leech Haemadipsa interrupta
Source: Parasitol Res. 2024 Nov 21;123(11):390. doi: 10.1007/s00436-024-08404-w (PMC11582220; doi:10.1007/s00436-024-08404-w)
Supplement: Supplementary file 1 — Supplementary file1 (PDF 2904 KB) [file 436_2024_8404_MOESM1_ESM.pdf]

Figure S1 Purification of Hint_V1 and Hint_V4

Step 1: Expression in *E. coli*

Hint_V1

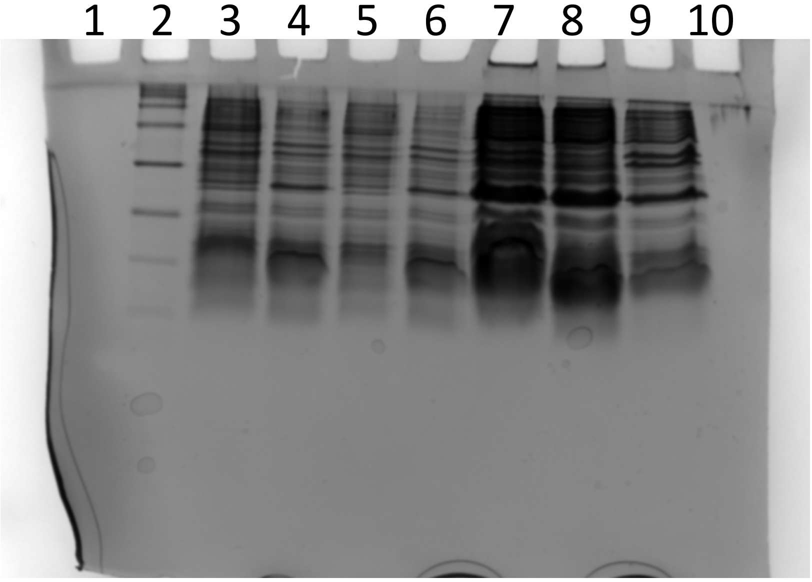

1 = -
2 = molecular weight standard
3 = flask 1 before induction
4 = flask 1 after induction
5 = flaskf 2 before induction
6 = flask 2 after induction
7 = cell lysate after sonication
8 = supernatant
9 = resuspended pellet
10= -

Hint_V4

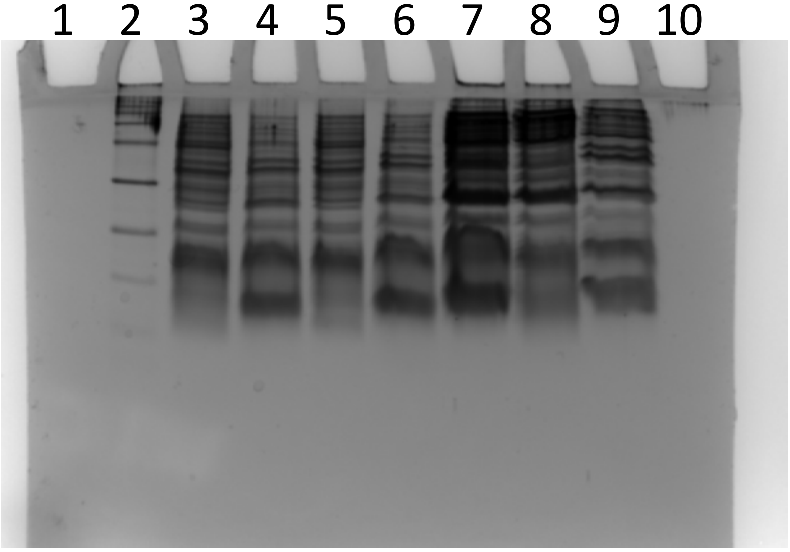

1 = -
2 = molecular weight standard
3 = flask 1 before induction
4 = flask 1 after induction
5 = flaskf 2 before induction
6 = flask 2 after induction
7 = cell lysate after sonication
8 = supernatant
9 = resuspended pellet
10= -

Purification of Hint_V1 and Hint_V4

Step 2: His-tag purification

Hint_V1

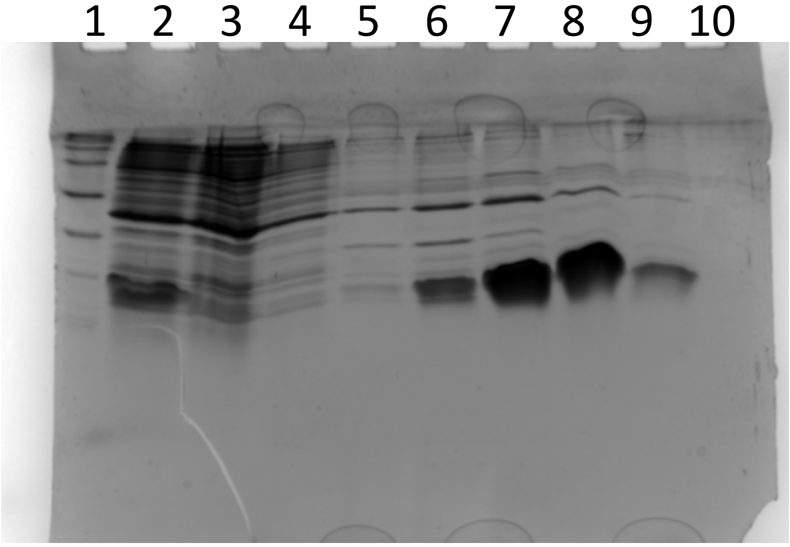

1 = molecular weight standard
2 = supernatant
3 = flow through
4 = fraction 5 mM imidazole
5 = fraction 20 mM imidazole
6 = fraction 50 mM imidazole
7 = fraction 100 mM imidazole
8 = fraction 200 mM imidazole
9 = fraction 500 mM imidazole
10= fraction strip buffer

Hint_V4

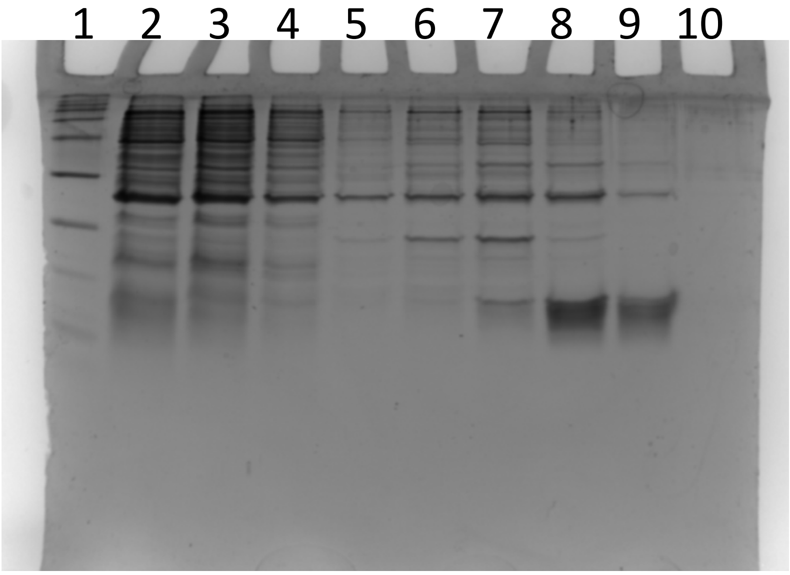

1 = molecular weight standard
2 = supernatant
3 = flow through
4 = fraction 5 mM imidazole
5 = fraction 20 mM imidazole
6 = fraction 50 mM imidazole
7 = fraction 100 mM imidazole
8 = fraction 200 mM imidazole
9 = fraction 500 mM imidazole
10= fraction strip buffer

Purification of Hint_V1 and Hint_V4

Step 3: Factor Xa treatment

Hint_V1

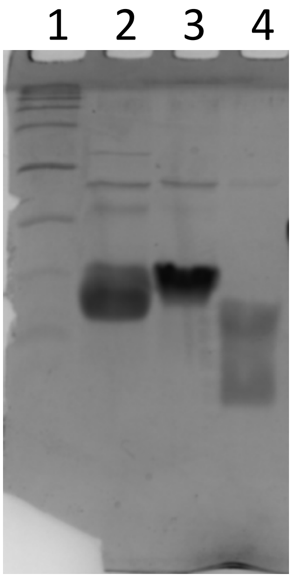

1 = molecular weight standard
2 = fraction 200 mM imidazole
3 = factor Xa treatment t 0 h
4 = factor Xa treatment t 60 h

Hint_V4

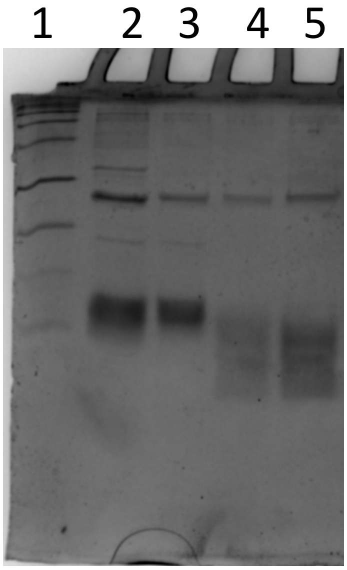

1 = molecular weight standard
2 = fraction 200 mM imidazole
3 = factor Xa treatment t 0 h
4 = factor Xa treatment t 60 h
5 = after concentration

Purification of Hint_DV1

Step 1: Expression *in E. coli*

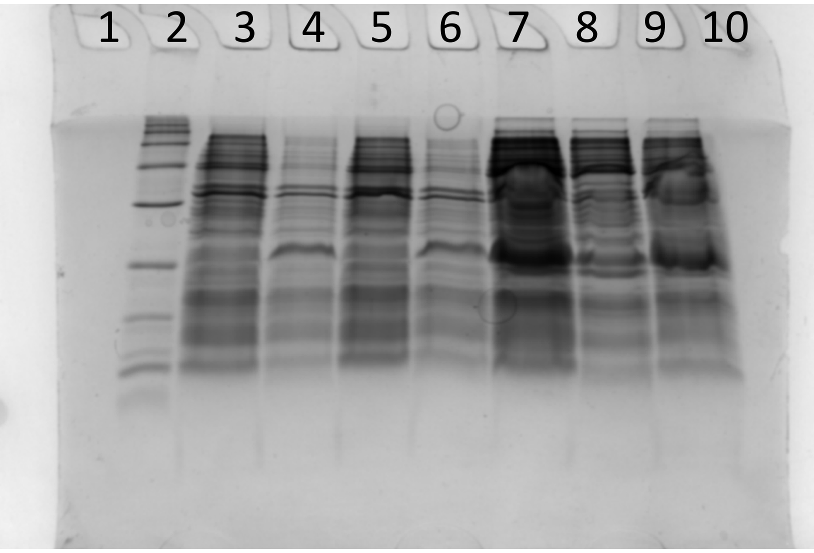

1 = -
2 = molecular weight standard
3 = flask 1 before induction
4 = flask 1 after induction
5 = flaskf 2 before induction
6 = flask 2 after induction
7 = cell lysate after sonication
8 = supernatant
9 = resuspended pellet
10= -

Step 2: His-tag purification

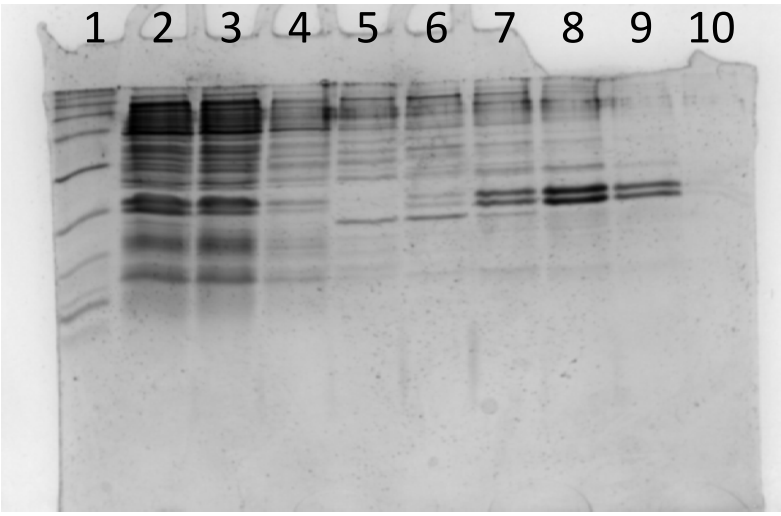

1 = molecular weight standard
2 = supernatant
3 = flow through
4 = fraction 5 mM imidazole
5 = fraction 20 mM imidazole
6 = fraction 50 mM imidazole
7 = fraction 100 mM imidazole
8 = fraction 200 mM imidazole
9 = fraction 500 mM imidazole
10= fraction strip buffer

Step 3: Factor Xa treatment

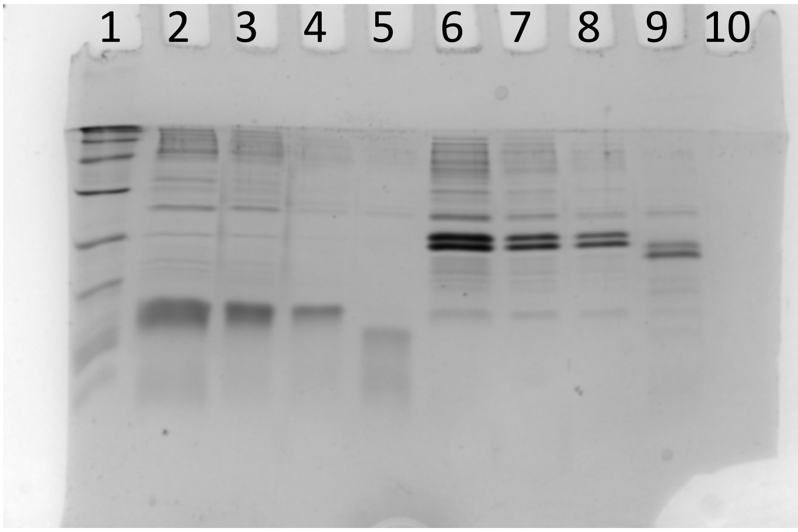

I Hint_DV1k I Hint_DV1 I

1 = molecular weight standard
2 = fraction 200 mM imidazole
3 = after dialysis
4 = factor Xa treatment t 0 h
5 = factor Xa treatment t 60 h
6 = fraction 200 mM imidazole
7 = after dialysis
8 = factor Xa treatment t 0 h
9 = factor Xa treatment t 60 h
10= -

Figure S2

MKSIILLLVFLQLTGIKR
RVSLS**C**WKKNTNLSK**C**N**C**GFET**C**YKGQK**C**VPP**RGD**DLDF**C**Y
PRYPN**C**TIFPKKSEQD**C**Y**C**GDHNVK**C**KPGQ**C**ILNKKIDFYE**C**LG
I**C**KKKKDSNER**C**I**C**GTKV**C**KKYQY**C**NKKKQ**C**KS
KNTTPKPSTTAKPKTKKKSRASPK

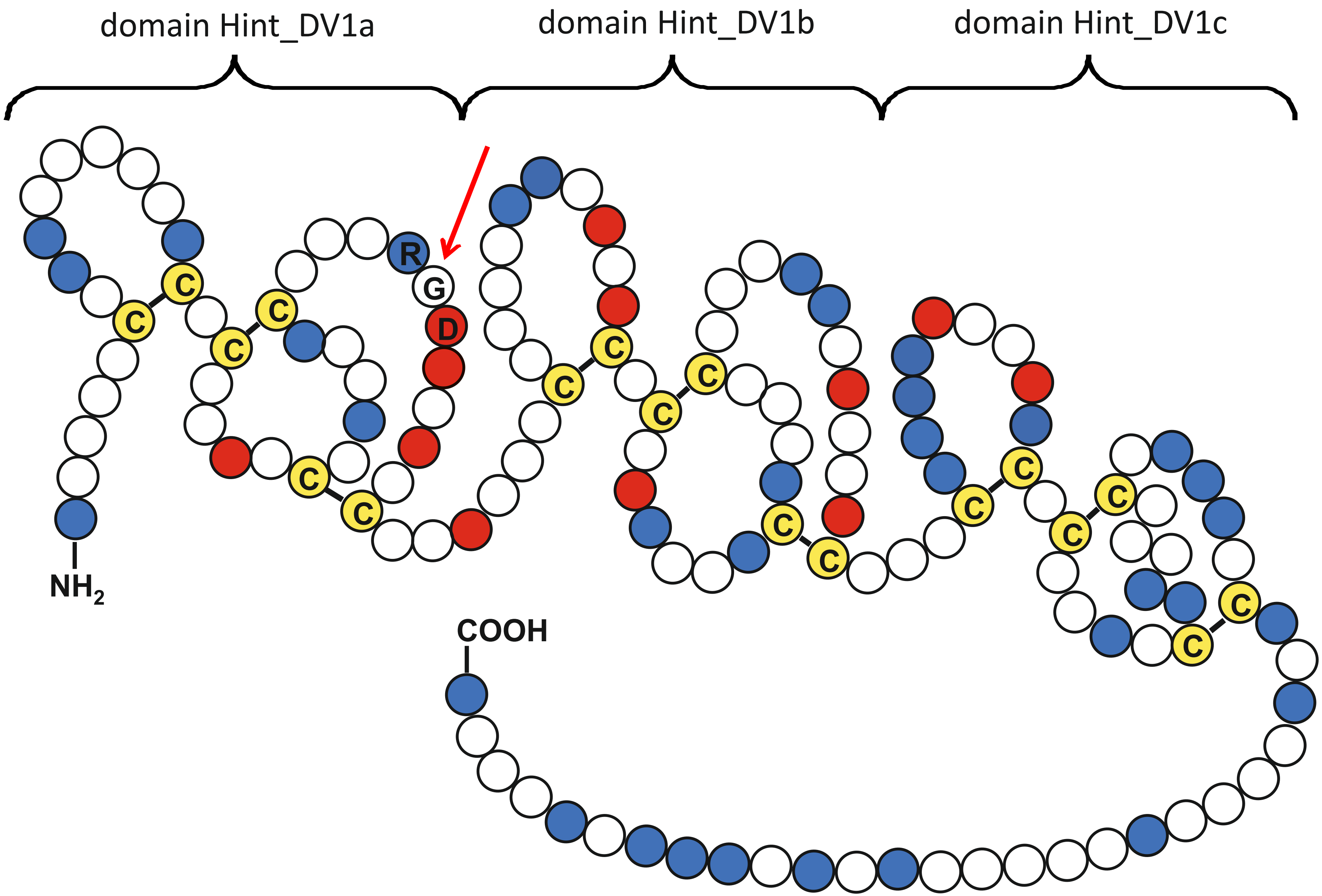

Table S1

List of Primers

Primers used for cloning in pQE30Xa:

Hint_V1:	Hint_fw1:	5`-GTG AGA TTT GGA ATG GGC AAT G-3`
	Hint_rev1:	5`-TAA AGC TTA TTT GTC TTC TTC ATC-3`
Hint_V2:	Hint_fw2a:	5`-GCG AGA ATT TTT CCA AAA TGT G-3`
	Hint_fw2b:	5`-AGA ATT TTT CCA AAA TGT GAG G-3`
	Hint_rev2:	5`-GAG AGC TCT CAA TAT TCA TGA TAC AAG C-3`
Hint_V3:	Hint_fw3:	5`-GTT AGA TTT GGA ATG GGC AAT G-3`
	Hint_rev3a:	5`-TTA AGC TTA TGA ATT ATT GAT TAT TAT TAG-3`
	Hint_rev3b:	5`-TTA AGC TTA TGA TTA ATT GAT TAT TAT TAG-3`
Hint_V4:	Hint_fw4:	5`-CAA ATA GAC GAT GCT CCC CCA TG-3`
	Hint_rev4:	5`-GAA AGC TTT TAT TTT ACA TCC CCT CTG-3`
Hint_DV1:	Hint_DV1_fw:	5`-CGT GTC TCA CTG TCC TGT TGG-3`
	Hint_DV1_rev:	5`-TTA AGC TTA TTT TGG AGA AGC TCT TG-3`

Table S2

	Hsyl	Hint_V1	Hint_V5	Hint_V2	Hint_V6	Hint_V7	Hint_V8	Hint_V9	Hint_V3	Hint_V4	Hint_V10	Hint_V11	Hint_V12
Hsyl	77	80%	76%	29%	37%	36%	32%	34%	54%	48%	45%	50%	41%
	0	87%	85%	41%	46%	44%	46%	42%	66%	64%	60%	61%	58%
	0	0%	0%	17%	17%	11%	26%	25%	10%	13%	16%	14%	17%
Hint_V1	62	77	94%	27%	41%	37%	32%	37%	56%	44%	43%	43%	40%
	67	0	96%	39%	49%	41%	45%	42%	67%	58%	55%	53%	52%
	0	0	0%	17%	17%	11%	26%	25%	10%	13%	16%	14%	17%
Hint_V5	59	73	77	26%	43%	39%	32%	37%	58%	44%	45%	45%	41%
	66	74	0	38%	50%	43%	45%	42%	69%	59%	58%	55%	52%
	0	0	0	17%	17%	11%	26%	25%	10%	13%	16%	14%	17%
Hint_V2	25	23	22	76	22%	19%	23%	26%	21%	21%	27%	26%	24%
	35	33	32	0	30%	32%	37%	33%	33%	34%	33%	34%	34%
	15	15	15	0	23%	17%	30%	39%	25%	21%	21%	20%	18%
Hint_V6	30	33	34	18	67	54%	32%	38%	39%	35%	42%	42%	35%
	37	39	40	25	0	67%	44%	41%	43%	43%	47%	46%	45%
	14	14	14	19	0	9%	13%	20%	26%	14%	17%	15%	16%
Hint_V7	29	30	31	16	40	72	33%	35%	34%	37%	33%	41%	36%
	35	33	34	26	49	0	43%	38%	39%	47%	42%	47%	42%
	9	9	9	14	7	0	21%	27%	20%	12%	15%	14%	14%
Hint_V8	26	26	26	19	22	25	60	40%	29%	32%	33%	38%	33%
	37	36	36	30	30	32	0	47%	40%	46%	52%	54%	45%
	21	21	21	24	9	16	0	16%	34%	23%	24%	22%	25%
Hint_V9	27	29	29	22	27	27	26	59	34%	32%	34%	37%	32%
	33	33	33	28	29	29	31	0	39%	40%	40%	40%	40%
	20	20	20	33	14	21	11	0	33%	22%	25%	24%	26%
Hint_V3	47	49	50	20	35	30	26	30	86	45%	46%	48%	42%
	57	58	60	31	38	35	36	34	0	56%	57%	55%	50%
	9	9	9	24	23	18	30	29	0	22%	24%	23%	25%
Hint_V4	39	36	36	18	27	29	25	24	41	74	54%	62%	48%
	52	47	48	29	33	37	35	30	51	0	72%	77%	63%
	11	11	11	18	11	10	18	17	20	0	2%	1%	7%
Hint_V10	37	35	37	23	32	26	25	26	42	40	72	75%	50%
	49	45	47	28	36	33	39	30	52	54	0	84%	58%
	13	13	13	18	13	12	18	19	22	2	0	1%	10%
Hint_V11	41	35	37	22	32	32	29	28	44	46	55	73	52%
	50	43	45	29	35	37	41	30	50	57	62	0	61%
	12	12	12	17	12	11	17	18	21	1	1	0	8%
Hint_V12	36	35	36	21	28	30	27	26	40	39	40	42	80
	50	45	45	30	36	35	36	32	48	51	47	49	0
	15	15	15	16	13	12	20	21	24	6	8	7	0

Table S3

	Mdec_Dec	Lmex_DV1	Lmex_DV2	Hint_DV1a	Hint_DV1b	Hint_DV1c
Mdec_Dec	39	26%	24%	30%	26%	20%
	0	34%	36%	38%	37%	32%
	0	9%	9%	9%	15%	32%
Lmex_DV1	11	39	74%	28%	24%	21%
	14	0	82%	38%	35%	39%
	4	0	0%	9%	15%	24%
Lmex_DV2	10	29	39	26%	22%	24%
	15	32	0	42%	33%	36%
	4	0	0	9%	15%	24%
Hint_DV1a	13	12	11	41	22%	26%
	16	16	18	0	33%	35%
	4	4	4	0	11%	23%
Hint_DV1b	12	11	10	10	44	26%
	17	16	15	15	0	31%
	7	7	7	5	0	28%
Hint_DV1c	9	9	10	11	12	33
	14	16	15	15	14	0
	14	10	10	10	13	0
